# Comprehensive analysis of the longan transcriptome reveals distinct regulatory programs during the floral transition

**DOI:** 10.1186/s12864-019-5461-3

**Published:** 2019-02-11

**Authors:** Dengwei Jue, Xuelian Sang, Liqin Liu, Bo Shu, Yicheng Wang, Chengming Liu, Yi Wang, Jianghui Xie, Shengyou Shi

**Affiliations:** 1 Key Laboratory of Tropical Fruit Biology (Ministry of Agriculture), South Subtropical Crops Research Institute, Chinese Academy of Tropical Agricultural Sciences, Zhanjiang, 524091 China; 2 College of Horticulture, South China Agricultural University, Guangzhou, 510642 China; 3 School of Advanced Agriculture and Bioengineering, Yangtze Normal University, Chongqing, 408100 China

**Keywords:** Longan, Floral transition, Perpetual flowering, Comprehensive transcriptome analysis

## Abstract

**Background:**

Longan (*Dimocarpus longan* Lour.) is an important fruit tree in the subtropical regions of Southeast Asia and Australia. Among the factors affecting *D. longan* fruit yield, the difficulty and instability of blossoming is one of the most challenging issues. Perpetual flowering (PF) is a crucial trait for fruit trees and is directly linked to production potential. Therefore, studying the molecular regulatory mechanism of longan PF traits is crucial for understanding and solving problems related to flowering. In this study, comparative transcriptome analysis was performed using two longan cultivars that display opposite flowering phenotypes during floral induction.

**Results:**

We obtained 853.72 M clean reads comprising 125.08 Gb. After comparing these data with the longan genome, 27,266 known genes and 1913 new genes were detected. Significant differences in gene expression were observed between the two genotypes, with 6150 and 6202 differentially expressed genes (DEGs) for ‘SJ’ and ‘SX’, respectively. The transcriptional landscape of floral transition at the early stage was very different in these two longan genotypes with respect to key hormones, circadian rhythm, sugar metabolism, and transcription factors. Almost all flowering-related DEGs identified are involved in photoperiod and circadian clock pathways, such as CONSTANS-like (*COL*), two-component response regulator-like (*APRRs*), gigantea (*GI*), and early flowering (*EFL*). In addition, the leafy (*LFY*) gene, which is the central floral meristem identity gene, may inhibit PF formation in ‘SJ’.

**Conclusion:**

This study provides a platform for understanding the molecular mechanisms responsible for changes between PF and seasonal flowering (SF) longan genotypes and may benefit studies on PF trait mechanisms of evergreen fruit trees.

**Electronic supplementary material:**

The online version of this article (10.1186/s12864-019-5461-3) contains supplementary material, which is available to authorized users.

## Background

Longan (*Dimocarpus longan* Lour.) is an important subtropical fruit tree that is widely grown in several subtropical and tropical countries [1]. As the country of origin, China has the highest longan production in the world [2, 3]. In general, longan varieties, such as the main cultivar ‘Shixia’ (‘SX’), exhibit seasonal flowering (SF). Floral bud induction in *D. longan* requires favorable conditions such as a period of low temperature (vernalization), suitable salinity and dry conditions. To obtain a stable high yield, longan flowering in the off-season is achieved through chemical treatment with potassium chlorate (KClO_3_) [4, 5], and region and tree variety greatly influence the induction effect. Therefore, research on the molecular regulatory mechanism of floral induction in longan is crucial for understanding and solving problems related to flowering. However, such knowledge regarding floral induction in longan is scarce because of its long generation time. The longan cultivar ‘Sijimi’ (‘SJ’) is a perpetual flowering (PF) genotype, which flowers and bears fruits throughout the year and does not require special external environmental conditions. Therefore, this cultivar is a good material for the study of longan flowering.

As an important developmental process in the plant life cycle, flowering is directly linked to production, regardless of when seeds or fruits are harvested [6], and thus flowering at an appropriate time is important for crop yield. In *Arabidopsis* and other model plants, the molecular mechanisms of flowering have been well established. There are at least five major flowering pathways in *Arabidopsis*, including photoperiod, autonomous, vernalization, gibberellin (GA), and aging pathways [7], that activate or inhibit floral transformation through a series of flower integron genes such as flowering locus T (*FT*), flowering locus C (*FLC*), and CONSTANS (*CO*) [8]. However, due to long generation times and complex genetic backgrounds, knowledge of the molecular genetics of flowering in perennials is scant compared to that in model plants [9]. For example, overexpression of *Arabidopsis LFY* in poplar (*Populus* spp.) resulted in early flowering in the transgenic lines [10], and flowers were observed within several months on transgenic lines overexpressing *FT1* and *FT2*, whereas the first flowers on wild-type trees were obtained after 5–10 years [11, 12]. In addition, ectopic expression of four alternative splicing forms of *Chrysanthemum FTL1* in *Arabidopsis* caused varying degrees of early flowering [13]. Moreover, longan *FT1* and *FT2* have been ectopically expressed in *Arabidopsis*, whereby *DlFT1-*and *DlFT2*-overexpressing lines showed early flowering and late flowering phenotypes, respectively. Interestingly, transgenic *Arabidopsis* lines overexpressing longan apetala1 (*AP1*) displayed a range of flowering time phenotypes [14]. Nonetheless, some flowering time-associated genes in *Arabidopsis* consistently fail to affect blossoming in trees. For example, overexpressed *MADS1* and *CO*, *LFY*, *AP1*, and agamous-like20 (*AGL20*) in poplar, the transgenic lines showed very rare and early floral onset or no flowers, suggesting the presence of different flowering regulatory mechanisms in perennials [15].

Because it extends the production period, PF is a crucial trait for fruit trees [16], and the genetic control of PF has been elucidated in several model plants. For instance, flowering 1 (*PEP1*), an ortholog of the floral repressor *FLC*, controls the PF trait in *Arabidopsis* [17], whereas PF in diploid strawberry and rose is due to a mutation in an ortholog of the floral repressor terminal flower 1 (*TFL1*) [16, 18]. Recent studies have also shown that the major *FaPFRU* locus, a nonortholog of *TFL1*, controls the PF trait in some strawberry cultivars [19, 20]. Regardless, multiyear delays in the onset of flowering and a long juvenile phase hamper studies of PF traits in perennials. Despite several studies on flowering genes in ‘SJ’ using RNA sequencing (RNA-Seq) analysis [4, 5], the molecular mechanism of PF traits in ‘SJ’ remains unknown. In this study, comparative transcriptome analysis during the floral induction process was performed using two longan cultivars (‘SJ’ and ‘SX’). Our aim is to clarify the genetic basis for different flowering capabilities between these two cultivars. The results of this study may provide valuable information regarding the molecular regulatory mechanisms of floral induction in two longan cultivars that differ in flowering time.

## Methods

### Plant materials

*D. longan* ‘SJ’ and ‘SX’ (nine-year-old trees), which display opposite flowering phenotypes, were grown at an experimental orchard of the South Subtropical Crops Research Institute of the Chinese Academy of Tropical Agricultural Science in Zhanjiang, China (110°16′ E, 21°10′ N). ‘SX’, one of the main varieties in China originating from Guangxi Province, exhibits SF traits, and floral bud induction requires a period of low temperature [21]. ‘SJ’, originating from China (Guangxi Province)/the Vietnam border region, exhibits PF traits, flowering and bearing fruits throughout the year under both high and low temperatures [22]. Previous molecular marker analyses have showed that ‘SJ’ has a close genetic relationship with longan cultivars of Guangxi Province and is clustered with Chinese cultivar groups including ‘SX’ [21, 23]. Three different types of ‘SJ’ and ‘SX’ apical buds were used in this study. Samples of the dormant stage (before the emergence of floral primordia) (T1) were collected on November 20, 2016; the apical bud at this stage is characterized by high hardness. Samples of floral primordia (red bud) (T2 stage) were collected on December 24, 2016; the apical bud at this stage is characterized by the appearance of red dot. Samples of the floral organ formation stage (T3) were collected on January 1, 2017; the apical bud at this stage is characterized by the appearance of the first inflorescence. Three biological replicates from three different trees were used for each sample. All samples were collected between 10 am and 12 am, placed immediately in liquid nitrogen and stored at − 80 °C until RNA-Seq and quantitative reverse transcription polymerase chain reaction (qRT-PCR).

### RNA extraction and Illumina sequencing

Total RNA was obtained using a quick RNA Isolation Kit (Huangyueyang, Beijing, China) according to the manufacturer’s instructions, and contaminant DNA was removed. The concentration and quality of the RNA were verified using an Agilent 2100 Bioanalyzer (Agilent Technologies, Palo Alto, CA, USA). Equal amounts of total RNA extracted from the three replicate plants at each flowering time comprised the cDNA library. Eighteen cDNA libraries (2 cultivars × 3 flowering times × 3 replicates) were constructed and sequenced using the Illumina HiSeq™ 2000 platform (Illumina Inc., CA, USA). Before assembly, adaptor sequences were removed from the raw reads. To obtain more reliable results, low-quality reads with over 50% bases were removed from each dataset. Those with quality scores of 5 or lower and/or over 10% bases unknown (N bases) were also removed. High-quality clean reads from 18 samples were mapped to the longan genome database [24] using SOAPaligner/soap2 [25].

### Differential gene expression (DEG) analysis

The level of unigene expression was normalized by calculating the reads per kilobase of exon model per million mapped reads (RPKM), as estimated using RSEM v1.2.15 [26]. To detect transcriptional changes in ‘SJ’ and ‘SX’ during flower induction, differential expression analysis was carried out using the DESeq R package (1.10.1) [27], which provides statistical routines for determining differential expression in digital gene expression data using a model based on negative binomial distribution. The resulting *p* values were adjusted using the Benjamini and Hochberg approach for controlling the false discovery rate (FDR). Genes with a minimal 2-fold difference in expression (|log_2_ Ratio| ≥ 1) and an adjusted *p*-value < 0.05 were considered differentially expressed [28]. To evaluate gene expression patterns during floral induction in ‘SJ’ and ‘SX’, expression pattern analyses were performed, and DEGs for ‘SJ’ and ‘SX’ were clustered into eight expression profiles using Short Time-series Expression Miner (STEM) version 1.3.8 [29]. The clustered profiles of DEGs with *p*-value < 0.05 were considered significantly different from the reference set for each genotype.

To verify biological significance, all DEGs were subjected to Gene Ontology (GO) enrichment analysis using Blast2GO (version 2.8) [30] and WEGO [31]. GO terms with and adjusted *p*-value (*p*.adjust) ≤ 0.05 were considered significantly enriched. DEGs were mapped to terms in the Kyoto Encyclopedia of Genes and Genomes (KEGG) database using BLASTX software [32]. Pathways with a q-value ≤0.05 were considered significantly enriched.

Transcription factors (TFs) were obtained from a previous study [24], and Mapman visualization [33] was performed to identify in the transcriptome data TFs that may play essential roles in regulating longan floral induction. Fragments per kilobase of the exon model per million mapped values (FPKM) were log_2_-transformed, and heat maps with hierarchical clustering were generated using the software Mev4.9.0 [34].

### Gene expression validation

Thirty-eight DEGs were selected to confirm the transcriptomic data through qRT-PCR analysis. Gene-specific primers were designed using Primer 3 software (Additional file 1). The three independent biological replicates for three flowering development times were mixed as T1, T2, and T3. First-strand cDNA was generated from purified total RNA using a PrimeScript™ RT reagent kit (Takara, Japan). Longan *actin* (Dlo_028674), which was used in our previous study, was selected as the reference gene [35]. qRT-PCR was conducted using the LightCycler® 480 Real-Time PCR System (Roche, Germany) and SYBR Green II PCR Master Mix (Takara, Japan). The amplification program was as follows: 95 °C for 5 min, followed by 40 cycles of 95 °C for 15 s and 60 °C for 1 min. Each reaction was performed in triplicate. The relative expression levels of the candidate genes were calculated using the 2^-∆∆Ct^ method [35]. Pearson’s correlation values between the RNA-Seq and qRT-PCR data of selected genes were calculated using “cor.test” in R version 3.3.

## Results

### Transcriptome assembly of sequencing reads

To identify genetic differences that may contribute to the PF traits of ‘SJ’ at the transcriptional level, comparative transcript profiling of ‘SX’ and ‘SJ’ flower buds from three different developmental stages was performed using RNA-Seq. The Illumina sequence data were deposited in the National Center for Biotechnology Information (NCBI) Sequence Read Archive (SRS2241241–SRS2241258). After filtering adaptor sequences and removing low-quality tags, 853.72 M clean reads were obtained, with 41,542,306–57,566,164 clean reads generated from the 18 samples and 125.08 Gb of sequence. The Q30 percentage (sequences with a sequencing error rate lower than 0.1%) was greater than 93%, and the average GC content was 44.70%. Among the total clean reads, approximately 32,322,773 (77.81%)–47,947,255 (83.29%) matched perfectly to the longan genome (Additional file 2) [24]. After merging these data, 27,266 (69.41%) known genes and 1913 new genes were identified (Additional file 3). These results indicate that the sequencing quality was sufficient for further analyses.

### Identification of DEGs between ‘SJ’ and ‘SX’ during floral induction

**Fig. 1:**
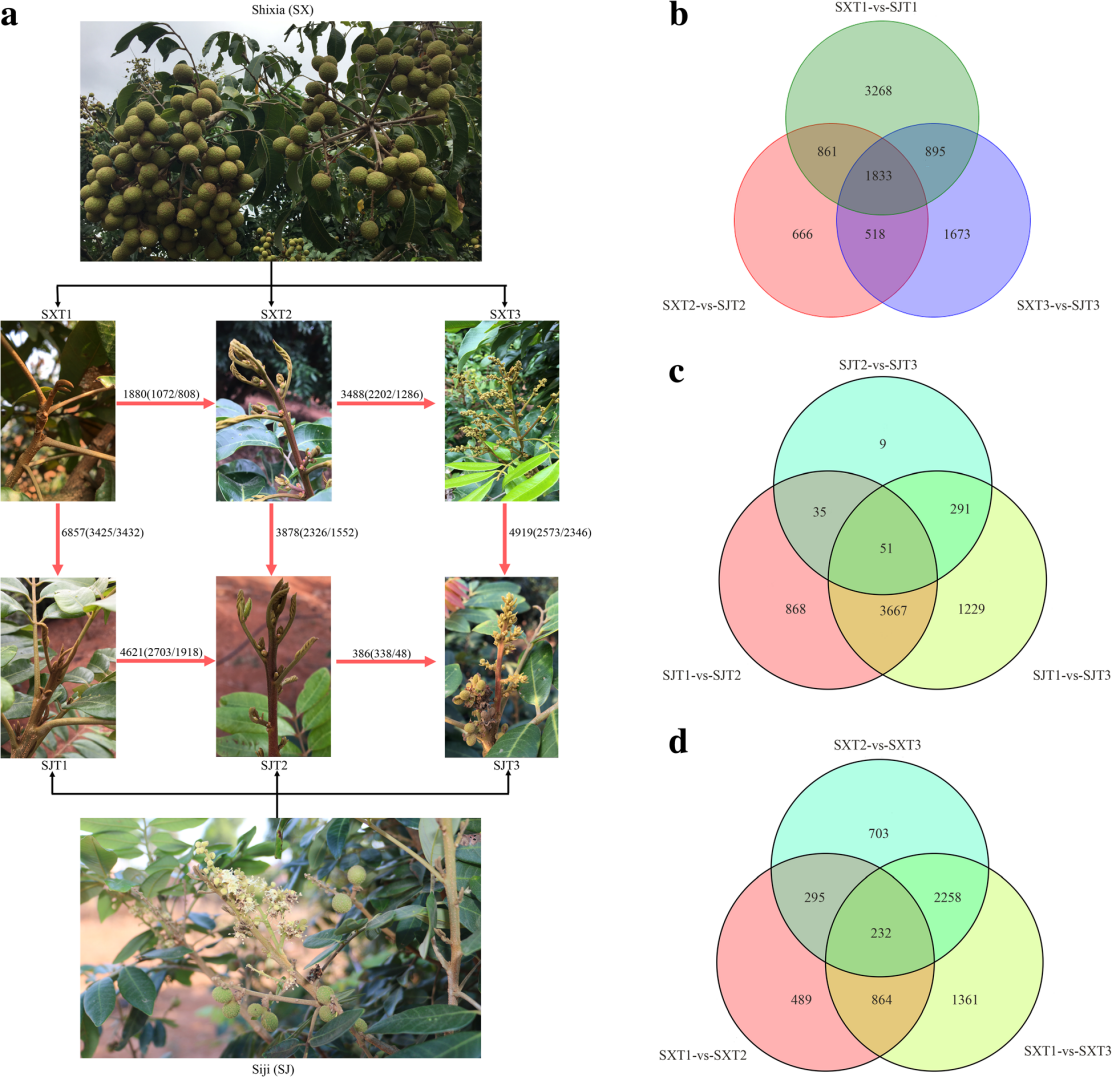
Different flowering phenotypes and number of differentially expressed genes during floral induction in ‘SJ’ and ‘SX’ longan. **a** Different flowering traits of ‘SJ’ and ‘SX’. ‘SJ’ longan blossoms continuously; both terminal and axillary shoots can differentiate into inflorescences, and flowers and fruits can be observed at the same time on one tree. T1 represents the dormant stage (before the emergence of floral primordia), T2 represents the emergence of floral primordium stage, and T3 represents the floral organ formation stage. The red arrows represent comparisons conducted in quantitative analyses. The numbers by the arrows denote the number differentially expressed genes for the specified comparison. **b** Venn diagram showing the number of DEGs between ‘SJ’ and ‘SX’ longan during the floral induction process. **c** Venn diagram showing the number of DEGs during floral induction in ‘SJ’. **d** Venn diagram showing the number of DEGs during floral induction in ‘SX’

DEGs were first identified through comparisons of the RPKM values for each gene of ‘SJ’ and ‘SX’ at different floral induction stages (SXT1-vs-SJT1, SXT2-vs-SJT2, and SXT3-vs-SJT3) with |log_2_ Ratio| ≥ 1 as the threshold of expression fold and FDR ≤ 0.05. A total of 9714 DEGs were detected in three pairwise stage comparisons: 6857 DEGs in T1 (SXT1-vs-SJT1), 3878 DEGs in T2 (SXT2-vs-SJT2) and 4919 DEGs in T3 (SXT3-vs-SJT3) (Fig. 1b and Additional file 4). Among the three pairwise stage comparisons, 1833 DEGs were common, indicating genetic differences between the two genotypes. GO and KEGG classifications were then performed to determine the functional significance of the transcriptional changes in each comparison. The significantly enriched GO terms (*p.adjust* < 0.05) are shown in Additional file 5. Regarding DEGs between ‘SJ’ and ‘SX’ at the dormant stage (T1), GO terms related to “cytoskeletal” and “microtubule” in the cellular component category, “microtubule-based process” and “oxidation-reduction process” in the biological process category, and those related to “binding” and “catalytic activity” in the molecular function category were significantly enriched. In the SXT2-vs-SJT2 comparison, only five significantly enriched GO terms in molecular function, including “kinase activity”, “phosphotransferase activity”, “chromatin binding”, “actin binding”, and “hexokinase activity”, were found. Regarding the SXT3-vs-SJT3 comparison, GO terms related to “sequence-specific DNA binding transcription factor activity”, “nucleic acid” and “oxidoreductase activity” in molecular function and those associated with “regulation of biological process” in biological process were significantly enriched.

In the SXT1-vs-SJT1, SXT2-vs-SJT2, and SXT3-vs-SJT3 comparisons, 813, 167, and 402 DEGs were mapped to 118, 72, and 98 KEGG pathways, respectively. Ten pathways were significantly enriched the SXT1-vs-SJT1 comparison (Additional file 6), including “starch and sucrose metabolism” (corrected *q*-value = 9.64 × 10^− 3^, 51 genes) and “plant hormone signal transduction” (corrected *q*-value = 1.73 × 10^− 2^, 106 genes). For the DEG comparison between ‘SJ’ and ‘SX’ at T2, pathways of “plant-pathogen interaction” (corrected *q*-value = 1.22 × 10^− 4^, 54 genes), “plant hormone signal transduction” (corrected *q*-value = 1.31 × 10^− 2^, 31 genes), and “circadian rhythm-plant” (corrected *q*-value = 2.26 × 10^− 2^, 8 genes) were significantly enriched. Finally, “plant hormone signal transduction” (corrected *q*-value = 8.37 × 10^− 4^, 67 genes) and “plant-pathogen interaction” (corrected *q*-value = 8.47 × 10^− 3^, 98 genes) were significantly enriched DEGs between ‘SJ’ and ‘SX’ at the T3 stage.

The results of GO and KEGG enrichment analyses suggested that the DEGs between ‘SJ’ and ‘SX’ at the dormant stage (T1) are mainly related to genes involved in plant basal metabolic processes; DEGs between ‘SJ’ and ‘SX’ at T2 and T3 are mainly involved in signal transduction and environmental adaptation. Interestingly, genes involved in circadian rhythm were only significantly enriched in the T2 stage, the emergence of the floral primordia, which is the signal for flower initiation.

### Quantitative transcriptomic changes during flower induction in ‘SJ’ and ‘SX’

Gene expression over the developmental course of flower induction was comparatively examined (T1-vs-T2, T2-vs-T3, and T1-vs-T3), revealing that 6150 and 6202 DEGs were significantly expressed in ‘SJ’ and ‘SX’, respectively (Fig. 1c, Additional file 7 and Additional file 8). Moreover, the distribution of changes between adjacent developmental stages was biased toward early floral induction in ‘SJ’, and 4621 (T1-vs-T2) and 386 (T2-vs-T3) DEGs were found (Fig. 1c), whereas the distribution of changes in ‘SX’ was biased toward the late floral induction stage, with 1880 (T1-vs-T2) and 3488 (T2-vs-T3) DEGs found. In addition, 5238 and 4715 DEGs were found in SJT1-vs-SJT3 and SXT1-vs-SXT3, respectively (Fig. 1d).

**Fig. 2:**
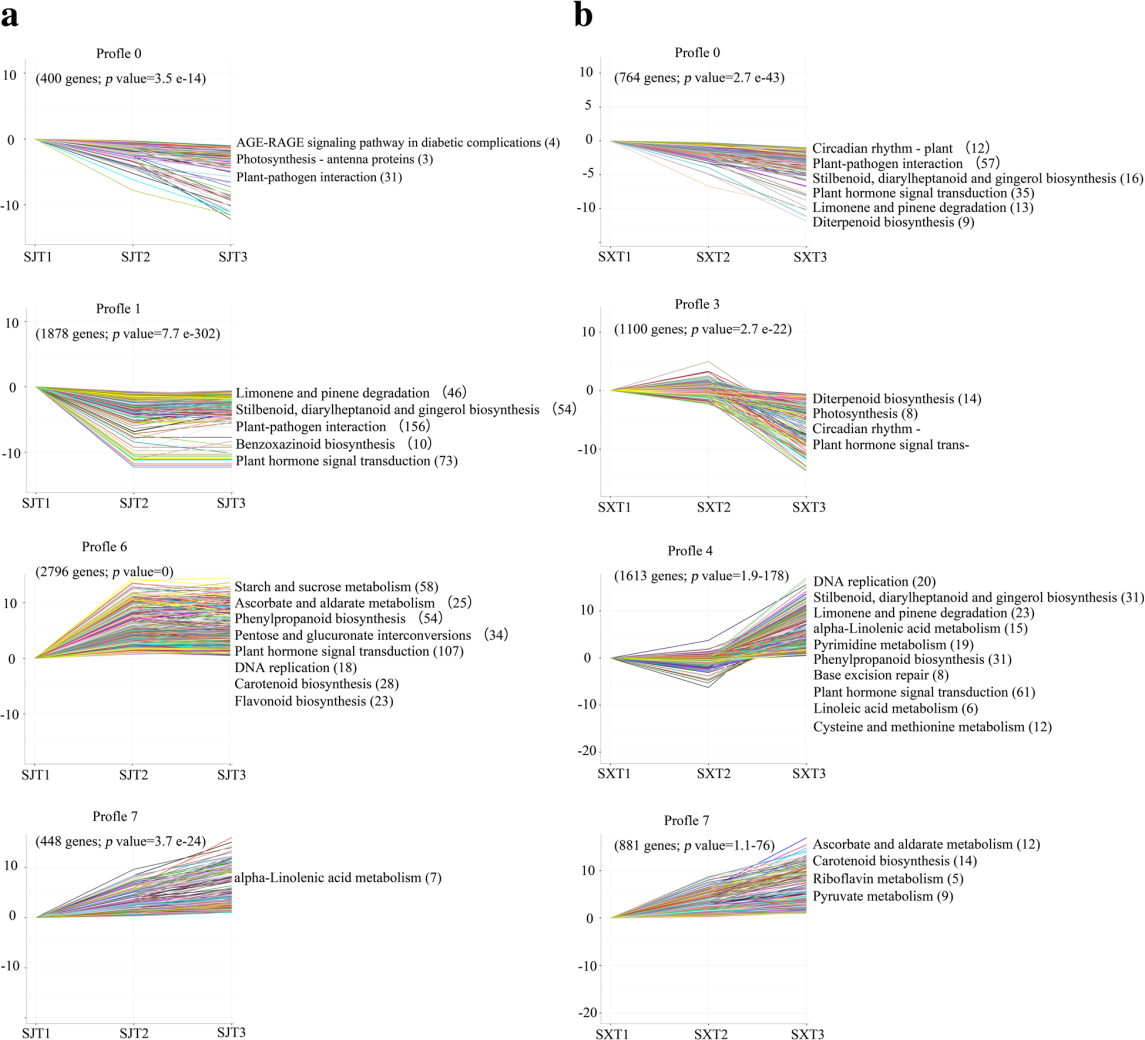
Trend analysis of DEGs with significant changes in expression profiles and KEGG pathway enrichment analysis for ‘SJ’ (**a**) and ‘SX’ (**b**). Genes coding for unknown products were not considered in the analysis. Enriched KEGG pathways are listed to the right of each profile

To evaluate the expression profiles, trend analyses were performed using STEM software, and the DEGs in ‘SJ’ and ‘SX’ clustered into 8 profiles. In ‘SJ’, 4 profiles had a significance of *p* < 0.05 (J0, J1, J6, and J7 with 400, 1878, 2796, and 448 DEGs, respectively) (Fig. 2a). GO functions and KEGG pathway enrichment in each main profile were also analyzed (Additional files 9 and 10). Profile 0 of ‘SJ’ had an overrepresentation of genes from processes associated with “photosynthesis”, “oxidation-reduction”, and “ATP/ADP binding”; significant pathways in this profile were “AGE-RAGE signaling pathway in diabetic complications” (*q*-value = 1.30 × 10^− 2^, 4 genes), “photosynthesis–antenna proteins” (*q*-value = 1.39 × 10^− 2^, 3 genes), and “plant-pathogen interaction” (*q*-value = 3.40 × 10^− 2^, 31 genes). Expression of the DEGs in profile 0 was decreased during the entire stage. Profile 1 showed more significantly enriched GO terms than did profile 0, and most of these 140 significantly enriched GO terms are related to “binging”, “catalytic activity”, “stress response”, and “regulation of biological process”. Moreover, genes involved in “limonene and pinene degradation” (*q*-value = 2.37 × 10^− 14^, 46 genes), “stilbenoid, diarylheptanoid and gingerol biosynthesis” (*q*-value = 5.52 × 10^− 14^, 54 genes), “plant-pathogen interaction” (*q*-value = 2.87 × 10^− 12^, 156 genes), “benzoxazinoid biosynthesis” (*q*-value = 7.32 × 10^− 4^, 10 genes), and “plant hormone signal transduction” (*q*-value = 1.56 × 10^− 2^, 73 genes) were significantly enriched. Gene expression decreased through T1 to T2 and remained low during the subsequent stage. In profile 6, there were 96 significantly enriched GO terms, with most DEGs being involved in “cellular biosynthetic”, “macromolecule biosynthetic”, “intracellular component biosynthetic” and other processes associated with cell proliferation and differentiation. The significantly enriched pathways of this profile were found to be “starch and sucrose metabolism” (*q*-value = 3.35 × 10^− 7^, 58 genes), “ascorbate and aldarate metabolism” (*q*-value = 4.81 × 10^− 7^, 25 genes), “phenylpropanoid biosynthesis” (*q*-value = 2.33 × 10^− 6^, 54 genes), “pentose and glucuronate interconversions” (*q*-value = 2.33 × 10^− 6^, 34 genes), “plant hormone signal transduction” (*q*-value = 5.88 × 10^− 6^, 107 genes), “DNA replication” (*q*-value = 1.10 × 10^− 5^, 18 genes), “carotenoid biosynthesis” (*q*-value = 2.10 × 10^− 4^, 28 genes), and “flavonoid biosynthesis” (*q*-value = 2.05 × 10^− 2^, 23 genes). The expression level of these genes in profile 6 peaked at T2 and remained high during T2 to T3. However, only two GO terms (“sequence-specific DNA binding transcription factor activity” and “nucleic acid binding transcription factor activity”) and one pathway (“alpha-linolenic acid metabolism”) were significantly enriched in profile 7, and gene expression in this profile increased during the entire stage.

In ‘SX’, 6202 DEGs clustered into 8 profiles. We also found 4 profiles with a significant *q*-value < 0.05 (Fig. 2b), with profiles X0, X3, X4, and X7 containing 764, 110, 1613, and 881 DEGs, respectively. Compared to ‘SJ’, profiles 3 and 4, in which the level of gene expression changed at the T2 to T3 transition, were significantly clustered in ‘SX’. Most of the DEGs involving “catalytic activity”, “molecular function regulator”, “regulation of biological process”, “organ development”, “immune response” and other processes involved in cell proliferation and differentiation were significantly enriched in profile 0. Moreover, pathways of “circadian rhythm-plant” (*q*-value = 4.45 × 10^− 4^, 12 genes), “plant-pathogen interaction” (*q*-value = 3.37 × 10^− 3^, 57 genes), “stilbenoid, diarylheptanoid and gingerol biosynthesis” (*q*-value = 9.26 × 10^− 3^, 16 genes), “plant hormone signal transduction” (*q*-value = 9.26 × 10^− 3^, 35 genes), “limonene and pinene degradation” (*q*-value = 9.26 × 10^− 3^, 13 genes), and “diterpenoid biosynthesis” (*q*-value = 9.26 × 10^− 3^, 9 genes) were overrepresented. DEGs involved in “photosynthesis” and “regulation of biosynthetic process” were significantly enriched in profile 3, as were pathways of “diterpenoid biosynthesis” (*q*-value = 3.37 × 10^− 5^, 14 genes), “photosynthesis” (*q*-value = 2.26 × 10^− 3^, 8 genes), “circadian rhythm” (*q*-value = 2.26 × 10^− 3^, 11 genes) and “plant hormone signal transduction” (*q*-value = 5.62 × 10^− 3^, 39 genes); gene expression remained at a high level at T1 to T2 and decreased during the subsequent stage. DEGs in profile 4 remained at a low level from T1 to T2 and increased during the subsequent stage. Overrepresentation of “DNA replication” (*q*-value = 3.77 × 10^− 10^, 20 genes), “stilbenoid, diarylheptanoid and gingerol biosynthesis” (*q*-value = 3.74 × 10^− 4^, 31 genes), “limonene and pinene degradation” (*q*-value = 2.55 × 10^− 3^, 23 genes), “alpha-linolenic acid metabolism” (*q*-value = 3.84 × 10^− 3^, 15 genes), “pyrimidine metabolism” (*q*-value = 3.84 × 10^− 3^, 19 genes), “phenylpropanoid biosynthesis” (*q*-value = 3.91 × 10^− 3^, 31 genes), “base excision repair” (*q*-value = 1.34 × 10^− 2^, 8 genes), “plant hormone signal transduction” (*q*-value = 1.48 × 10^− 2^, 61 genes), “linoleic acid metabolism” (*q*-value = 1.66 × 10^− 2^, 6 genes), and “cysteine and methionine metabolism” (*q*-value = 2.78 × 10^− 2^, 12 genes) were enriched in this profile. In turn, profile 7 contained more DEGs significantly enriched in “catalytic activity”, “single-organism process” and cell wall association function categories, and significantly enriched pathways were “carotenoid biosynthesis” (*q*-value = 5.17 × 10^− 4^, 12 genes), “ascorbate and aldarate metabolism” (*q*-value = 3.39 × 10^− 3^, 14 genes), “pyruvate metabolism” (*q*-value = 2.04 × 10^− 2^, 5 genes), and “riboflavin metabolism” (*q*-value = 2.58 × 10^− 2^, 9 genes) (Tables S8 and S9).

Compared to ‘SX’, gene regulation at the transcriptional level in ‘SJ’ was more active with regard to the period of dormancy release (T1 to T2 transition), whereas the major transcriptional regulation of ‘SX’ occurred at the period of floral organ formation (T2 to T3 transition). DEGs in starch and sucrose metabolism pathways were only significantly enriched in profile 6 of ‘SJ’, with increased expression at T1 to T2 for *beta-glucosidase 40* (Dlo_026115.1), *sucrose synthase* (Dlo_019926.1), and *endoglucanase 17* (Dlo_023528.1) (Additional file 7). In addition, DEGs associated with plant hormone signal transduction were significantly clustered in profiles 1 and 6 in ‘SJ’, which were up- or downregulated, respectively, during T1 to T2. Although some genes clustered in ‘SX’ profiles 3 and 4, they were upregulated or downregulated during the T2 to T3 transition, including DELLA protein GAI (Dlo_019465.1), AUX_IAA domain-containing protein (Dlo_021779.1), and auxin-induced protein 22C-like (Dlo_004907.3) (Additional file 8). Furthermore, the pathway related to photosynthesis clustered in different profiles in ‘SJ’ and ‘SX’, and the circadian rhythm pathway was only significantly enriched in profile 3 of ‘SX’. The results indicate that these pathways may play crucial roles in the formation of different flowering traits between the two genotypes.

### DEGs related to starch and sucrose metabolism

**Fig. 3:**
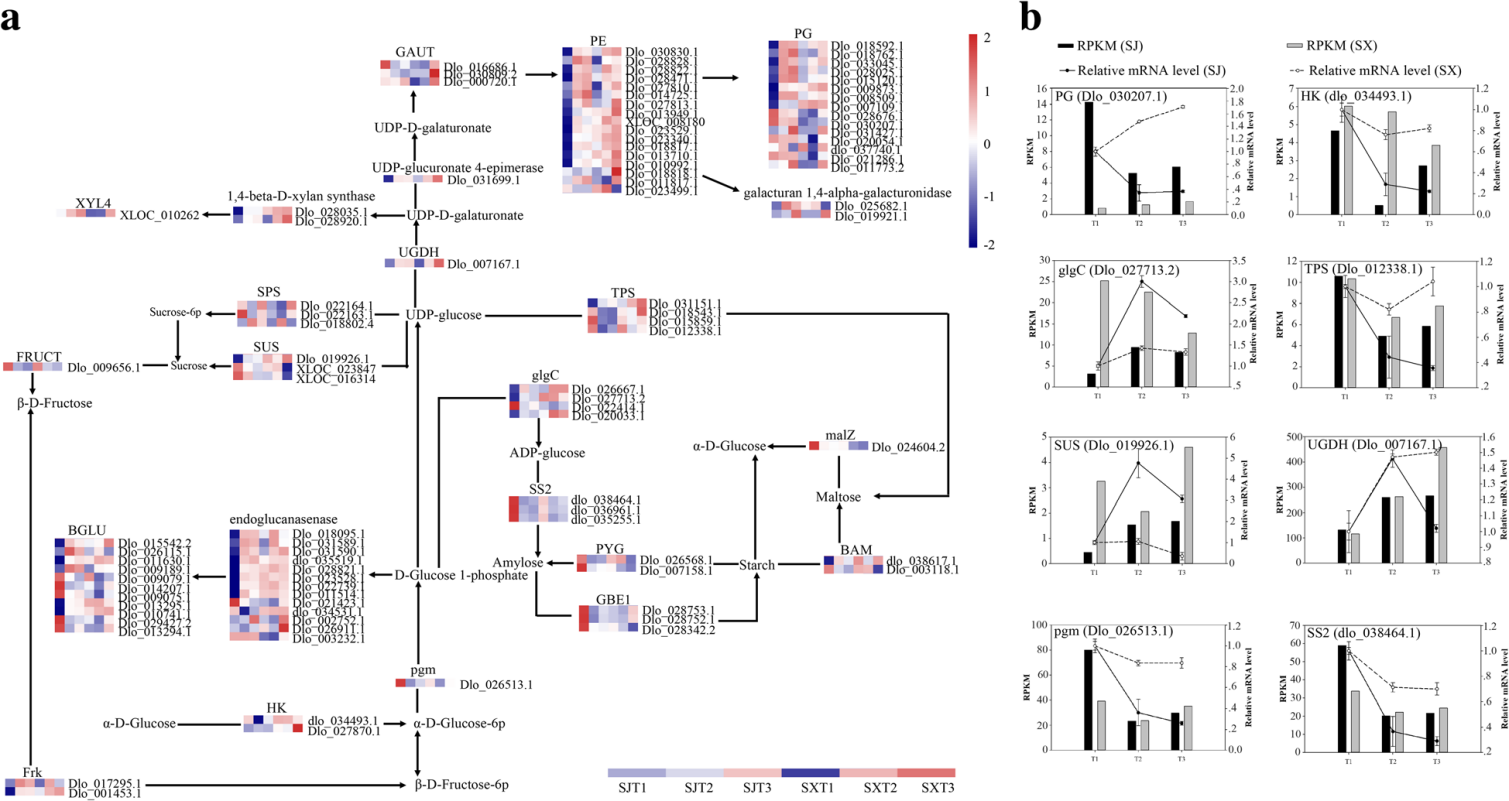
Expression profiles of sugar-related genes and qRT-PCR identification of sugar-related gene expression levels in flower buds during the floral induction process in ‘SJ’ and ‘SX’ longan. **a** Heat map of the comparative expression levels of sugar-related genes. Data for gene expression levels were normalized by the Z-score. Red and blue indicate up- and downregulated genes, respectively. **b** qRT-PCR identification of sugar-related gene expression levels in buds. The bar and line graphs are derived from RNA-Seq and qRT-PCR data, respectively. Values are the means of three replicates ± SE

As the starch and sucrose metabolism pathway was only significantly enriched in profile 6 in ‘SJ’ and the SXT1-vs-SJT1 comparison, the 97 DEGs (Additional file 11) in this pathway were further analyzed. Compared to ‘SX’, 33 genes were significantly upregulated during the T1 to T2 transition in ‘SJ’, including *pectinesterase*, *galacturan 1,4-alpha-galacturonidase*, *UDP-glucuronate 4-epimerase*, *1,4-beta-D-xylan synthase*, *glgC*, *beta-amylase*, *sucrose synthase* (Dlo_019926.1), *beta-glucosidase*, *fructokinase*, and *endoglucanase* (Fig. 3a). Moreover, 27 genes were significantly downregulated, such as *GAUT* (Dlo_016686.1), *TPS* (*trehalose 6-phosphate synthase*), *starch synthase*, *PYG*, *alpha-glucosidase*, *sucrose-phosphate synthase*, *HK* (*hexokinase*), and *pgm* (*phosphoglucomutase*). However, only 4 genes (*1,4-beta-D-xylan synthase*, Dlo_028920.1; *glucose-1-phosphate adenylyltransferase*, Dlo_020033.1; *sucrose-phosphate synthase*, Dlo_018802.4; *UGDH*, Dlo_007167.1) and 3 genes (*polygalacturonase*, Dlo_020054.1 and dlo_037740.1; *beta-amylase*, dlo_038617.1) were significantly upregulated or downregulated at the same stage in ‘SX’. There were 13 and 2 common genes significantly upregulated or downregulated, respectively, in these two cultivars. No genes in ‘SJ’ were significantly up- or downregulated during the T2 to T3 transition. Although 23 and 9 genes were significantly upregulated and downregulated at the same stage in ‘SX’, 4 genes common to these two cultivars were significantly upregulated.

### Differential gene expression in hormone signaling pathways

**Fig. 4:**
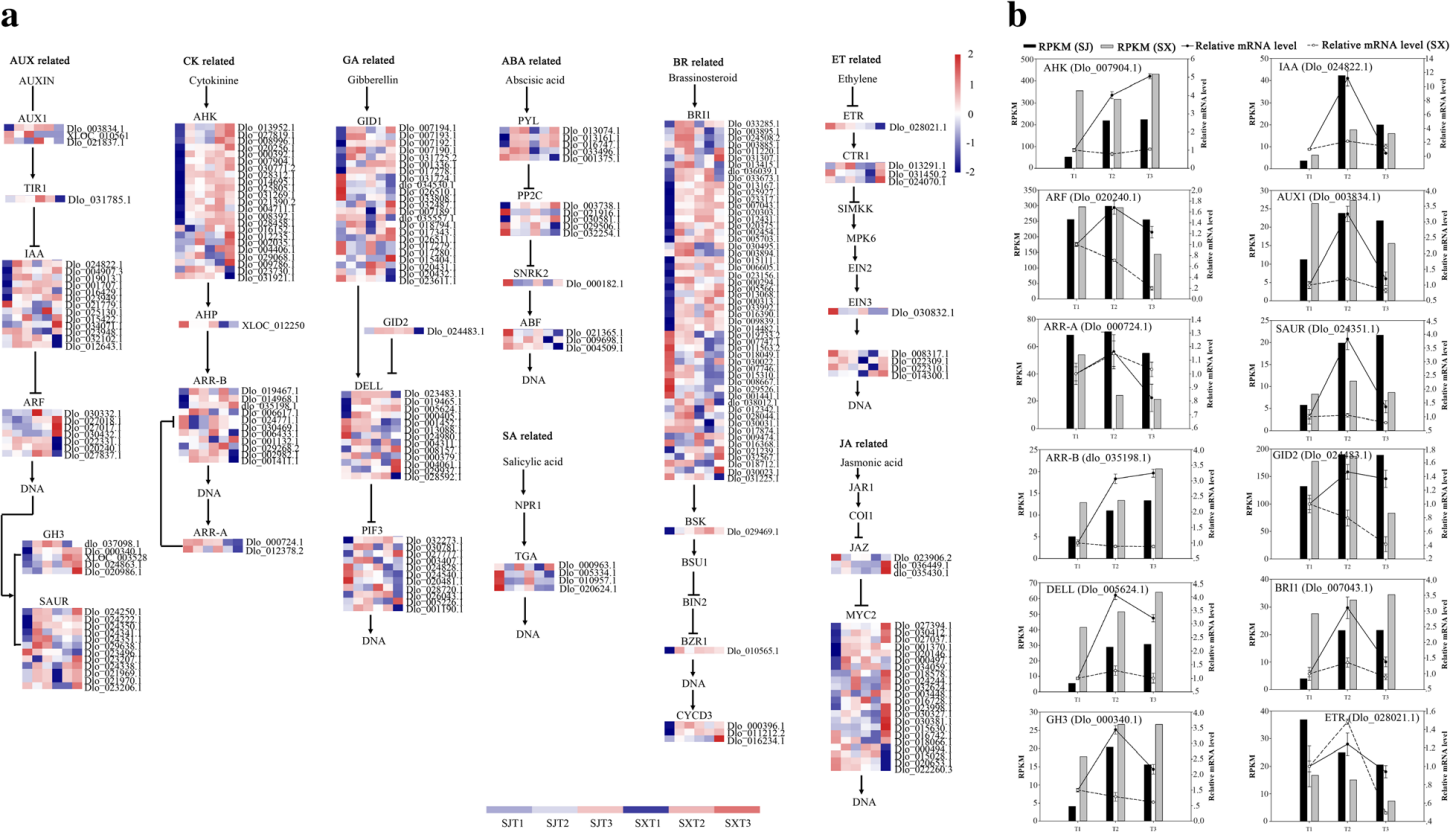
Expression profiles of hormone-related genes and qRT-PCR identification of hormone-related gene expression levels in flower buds during the floral induction process in ‘SJ’ and ‘SX’ longan. **a** Heat map of the comparative expression level of hormone-related genes. Data for gene expression levels were normalized by the Z-score. Red and blue indicate up- and downregulated genes, respectively. **b** qRT-PCR identification of hormone-related gene expression levels in buds. The bar and line graphs are derived from RNA-Seq and qRT-PCR data, respectively. Values are the means of three replicates ± SE

The plant hormone signal transduction pathway was found to be enriched in three comparisons between ‘SJ’ and ‘SX’ and in different profiles in the two cultivars during floral induction. To identify key DEGs regulating PF traits, the DEGs involved in hormone signaling were further analyzed using Mapman (Fig. 4).

In this study, 237 DEGs related to hormone signaling pathways were identified (Additional file 12). Compared to ‘SX’, DEGs related to hormone signaling pathways in ‘SJ’ were predominantly enriched in the early flower induction stage, with 89 and 28 DEGs displaying significant upregulation or downregulation, respectively, through T1 to T2. Among these 117 DEGs, 16 associated with the auxin pathway, including *AUX1* (Dlo_003834.1), five auxin-responsive genes, two indole-3-acetic acid-amido synthetase (*GH3*) genes, and six *SAUR* family genes, showed upregulation, and one *SAUR32-like* gene (Dlo_023207.1) and one auxin-responsive gene (Dlo_021779.1) showed downregulation; 16 genes related to the CK pathway were upregulated, and 3 genes were downregulated, including *AHK* and *ARR-A*. Moreover, 11 genes related to the GA pathway were upregulated, including 7 gibberellin receptor GID1 and 4 *DELLA* genes, and 3 *GID1* and 2 *DELLA* genes were downregulated. Two *PP2C* and 2 *PYL* genes associated with the ABA pathway were upregulated, and 2 *PP2C*, 1 *SRK2,* and 1 *ABF* were downregulated. In addition, 33 and 7 DEGs involved in the brassinosteroid signaling pathway exhibited upregulation or downregulation, including *BRI1*, *BSK*, *BRZ1/2* and *CYCD3*; one *CTR1* (Dlo_013291.1) and one *ERF1* (Dlo_022309.1) related to the ET pathway showed upregulation, and one *EIN3* (Dlo_030832.1) and one *ERF1* (Dlo_008317.1) displayed downregulation. Eight and two DEGs related to the JA pathway were upregulated or downregulated, respectively, during T1 to T2. Three TGA TFs related to the SA pathway displayed downregulation in ‘SJ’ during T1 to T2. In ‘SJ’, only *AUX1* (XLOC_010561) and *PIF3* (Dlo_027777) were upregulated or downregulated during T2 to T3. Compared to ‘SJ’, most DEGs related to hormone biosynthesis and signaling pathways in ‘SX’ were enriched in the late floral induction stage: 43 DEGs were upregulated or downregulated during T1 to T2. In contrast, 92 DEGs displayed upregulation or downregulation in ‘SX’ through T2 to T3 compared to ‘SJ’. Among these 43 DEGs, 22 displayed significant upregulation, including one *IAA* gene (Dlo_025130.1), three *SRURs* and two *GH3* genes of the Aux-mediated signaling pathway, 5 *GID1* and two *DELLA* genes involved in the GA-mediated signaling pathway, one *AHK* gene (Dlo_004406) related to the CK pathway, and one *PYL*, three *BRI1* and one *MYC2* (Dlo_003448.1) involved in the ABA, brassinosteroid and JA-mediated signaling pathways, respectively. Twenty-one DEGs were significantly downregulated during T1 to T2 compared to ‘SJ’, including an auxin influx carrier family protein and ARF involved in the Aux-mediated signaling pathway, two-component response regulator ARR-A and ARR-B family proteins related to the CK pathway, and *GID1*, *DELLA*, *ABF* and *BRI1* involved in the GA, ABA, and brassinosteroid-mediated signaling pathways. Among the 92 DEGs significantly upregulated or downregulated through T2 to T3 in ‘SX’, 51 were upregulated and 37 downregulated. These genes are related to auxin, GA, CK, ABA, ET and JA-mediated signaling pathways; the SA pathway was not represented in this case.

### Identification of flowering-related DEGs during flower induction

**Fig. 5:**
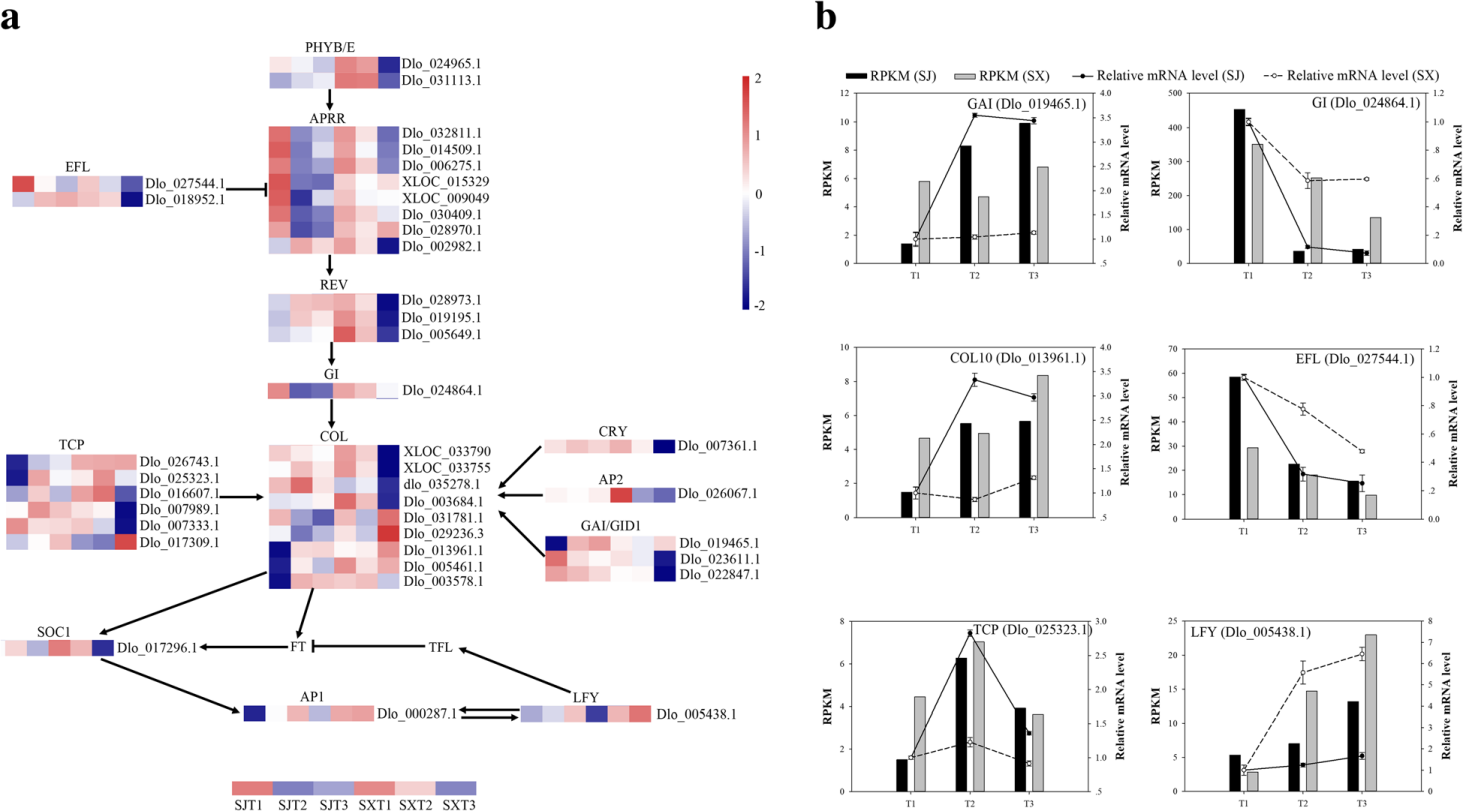
Expression profiles of flower-related genes and qRT-PCR identification of flower-related gene expression levels in flower buds during the floral induction process in ‘SJ’ and ‘SX’ longan. **a** Heat map of the comparative expression level of flower-related genes. Data for gene expression levels were normalized by the Z-score. Red and blue indicate up- and downregulated genes, respectively. **b** qRT-PCR identification of flower-related gene expression levels in buds. The bar and line graphs are derived from RNA-Seq and qRT-PCR data, respectively. Values are the means of three replicates ± SE

A hierarchical heat map was constructed to comparatively analyze the 39 flowering-related genes (Additional file 13) identified in this study (Fig. 5). Among these 39 DEGs, in ‘SJ’, 6 were only significantly upregulated during T1 to T2, including three *CONSTANS-like* family genes, *GAI* (Dlo_019465.1) and two *TCP* genes, and seven were only significantly downregulated during T1 to T2, including an *APRR* family gene, *EFL* (Dlo_027544.1) and *GI* (Dlo_024864.1). In contrast, only one gene (*LEAFY*, Dlo_005438.1) showed significant upregulation during T1 to T2 in ‘SX’, whereas *AP2* (Dlo_000287.1) and *CONSTANS-like 14* (Dlo_031781.1) displayed significant upregulation during T1 to T2. The remaining genes were significantly and differentially expressed during T2 to T3 in ‘SX’, including phytochrome (*phy*), embryonic flower 1, *SOC1–1*, *CONSTANS-like*, *TCP*, *REVEILLE*, *GID*, *FKF1* and cryptochrome (*cry*). Most of these flowering-related DEGs are associated with photoperiod and the circadian clock pathways, such as the *COL* gene, *APRRs*, *FKF1*, phytochrome, and TF TCPs. Moreover, only one gene (*LEAFY*, Dlo_005438.1) involved in the floral meristem pathway was significantly upregulated during T1 to T2 in ‘SX’, yet it showed no apparent change during the flower induction process in ‘SJ’. However, *TFL1* and *FLC*, which controls the PF trait in *Arabidopsis* and strawberry, did not show differential expression during the floral induction process in our study. These results indicate that photoperiod and circadian clock and floral meristem pathways have significant roles in regulating PF trait formation in longan.

### Identification of transcription factor-related DEGs during flower induction

**Fig. 6:**
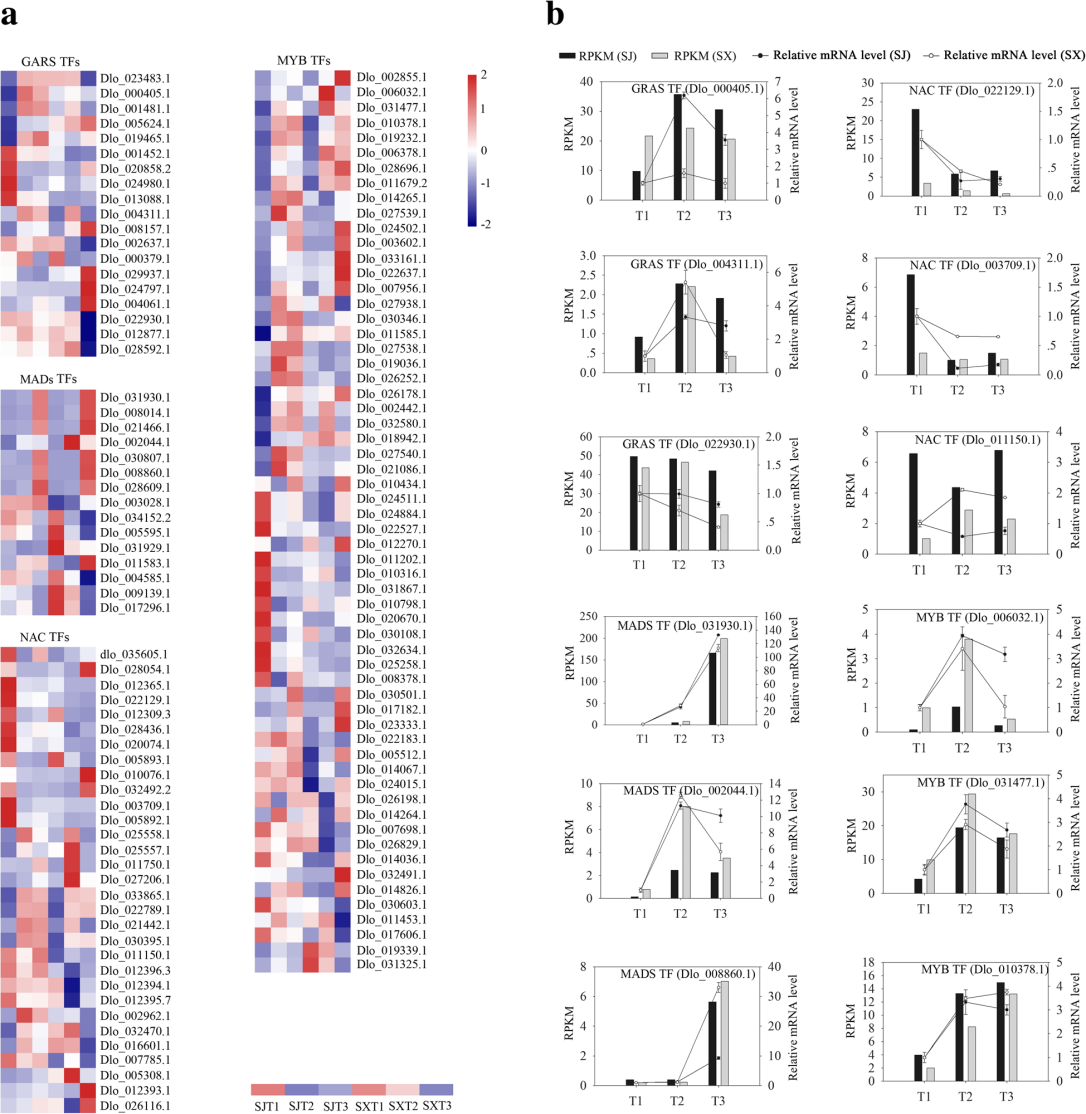
Expression profiles of transcription factor-related genes and qRT-PCR identification of transcription factor-related gene expression levels in flower buds during the floral induction process in ‘SJ’ and ‘SX’ longan. (**a**) Heat map of the comparative expression level of transcription factor-related genes. Data for gene expression levels were normalized by the Z-score. Red and blue indicate up- and downregulated genes, respectively. **b** qRT-PCR identification of transcription factor-related gene expression levels in buds. The bar and line graphs are derived from RNA-Seq and qRT-PCR data, respectively. Values are the means of three replicates ± SE

**Fig. 7:**
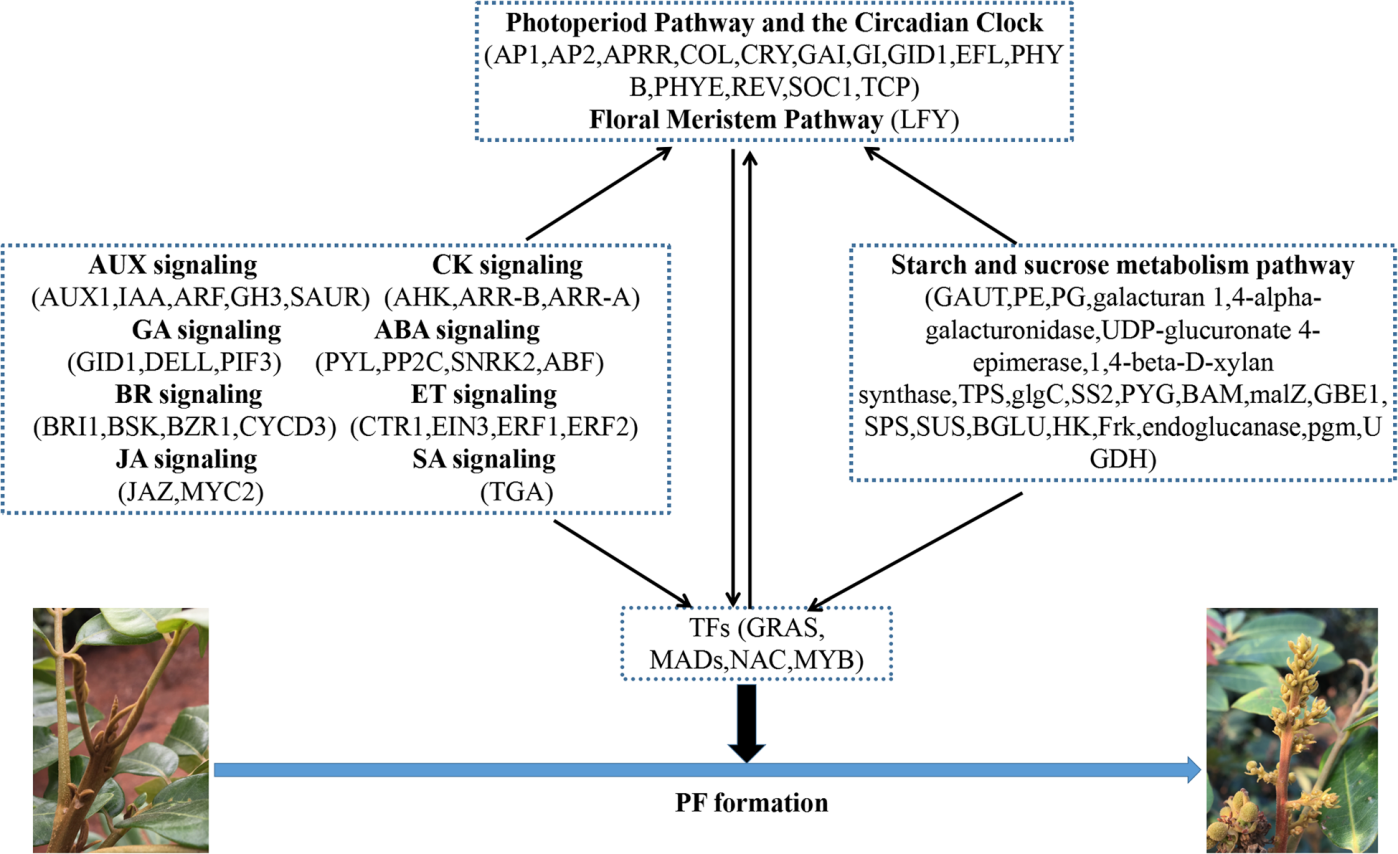
Summary of transcriptional-level regulation of longan PF trait formation

In the present study, we found many DEGs encode TFs such as GRAS, MADs, NAC and MYB TFs, which are involved in the regulation of floral transition [36–39]. As shown in Fig. 6, 19 GRAS TFs displayed differential expression during flower induction in ‘SJ’ and ‘SX’, including eight genes that were significantly up- or downregulated during T1 to T2 in ‘SJ’ and ten with differential expression during flower induction in ‘SX’. In addition, one GRAS family gene (Dlo_001452.1) showed significant downregulation in these two cultivars (Additional file 14). Fifteen DEGs are MADs TFs, including one gene (Dlo_021466.1) that displayed significant upregulation during T1 to T2 in ‘SJ’, one gene (Dlo_002044.1) that was significantly downregulated during T2 to T3 of ‘SX’, eight genes that displayed differential expression during flower induction in ‘SX’, and two genes (Dlo_031930.1 and Dlo_008014.1) that exhibited a constant increase in both cultivars (Additional file 14). Thirty-one NAC-like family members displayed differential expression during flower induction in ‘SJ’ and ‘SX’, including five (dlo_035605.1, Dlo_010076.1, Dlo_032492.2, Dlo_003709.1 and Dlo_005892.1) significantly downregulated genes during T1 to T2 in ‘SJ’, and seven (Dlo_028054.1, Dlo_012365.1, Dlo_022129.1, Dlo_012309.3, Dlo_028436.1, Dlo_020074.1 and Dlo_005893.1) common genes that were significantly downregulated during T1 to T2 in both cultivars; the remaining 19 genes displayed differential expression during flower induction in ‘SX’ (Additional file 14). Moreover, MYB family genes displayed differential expression during flower induction in ‘SJ’ and ‘SX’, including 19 and eight with significant downregulation during T1 to T2 in ‘SJ’, 17 with differential expression during flower induction in ‘SX’, and 34 with differential expression during flower induction in both cultivars (Additional file 14). DEGs likely involved in the longan PF trait are summarized in Fig. 7.

### Verification of RNA-Seq results by qRT-PCR

To further validate the RNA-Seq results, 38 DEGs involved in sugar metabolism, hormone signal transduction, photoperiod and circadian clock pathways were selected for parallel qRT-PCR-based expression analysis in ‘SJ’ and ‘SX’ (Figs. 3b, 4b, 5b, and 6b). The qRT-PCR data were in agreement with the RNA-Seq data, confirming the accuracy of our transcriptomic analysis (Pearson correlation coefficient R^2^ = 0.9782; Additional file 15).

## Discussion

It is important to study transcriptional regulation and its effect on phenotype [40]. Although several studies have examined flowering genes in ‘SJ’ using RNA-Seq technology [4, 5], the molecular mechanism underlying PF traits in ‘SJ’ remains unknown. Thus, to clarify the genetic basis for PF formation in ‘SJ’, comparative transcriptome analysis was performed in this study. A total of 853.72 M clean reads comprising 125.08 Gb were obtained. After comparing these data with the longan genome, 27,266 known genes and 1913 new genes were identified [24]. To monitor the transcriptional changes in these two longan cultivars (‘SJ’ and ‘SX’) during the floral induction process, DEGs were compared between these cultivars during flower induction. A total of 6150 DEGs were identified during the floral induction process in ‘SJ’, including 4621 in the T1 to T2 transition and 386 in the T2 to T3 transition; in ‘SX’, 1880 DEGs in the T1 to T2 transition and 3488 in the T2 to T3 transition were found (Fig. 1). These results show that DEGs related to PF were mainly enriched in the T1 to T2 transition, which is consistent with a previous study suggesting that red buds (T2 stage) are a signal of longan floral initiation [41]. We found that DEGs in the starch and sucrose metabolism pathway were only significantly enriched in profile 6 of ‘SJ’, which showed increased expression at the T1 to T2 transition, and that DEGs associated with plant hormone signal transduction, which were upregulated or downregulated during T1 to T2, were significantly clustered in profiles 1 and 6 in ‘SJ’ (Fig. 2). These results suggest that sugar and hormone pathways may play crucial roles in longan PF trait formation. Similar results have been reported for rose species [8].

The transition from dormancy to active bud growth requires sufficient energy [42]. As the main source of energy, carbohydrates are key for several plant developmental stages, including flower induction [43]. For example, the rate of leaf sucrose export increases during floral induction in *Arabidopsis* [44]. Similarly, increases in stored carbohydrates promote floral initiation in several horticultural trees. In fact, girdling, which has been reported to increase levels of stored carbohydrates, promotes flowering intensity in olive [45], lychee [46], and citrus [47]. In contrast, the contents of soluble sugar and starch in the buds of PF rose were higher than those in the buds of SF rose [8]. These results indicate that a suitable concentration of carbohydrates may be associated with floral induction and PF traits in plants. Consistent with such changes in carbohydrate contents, expression of genes related to starch and sucrose metabolism also displayed differential expression during floral induction. For instance, levels of *TPS1*, *fructose-1,6-bisphosphatase I* (*FBP*), and *sucrose synthase* (*SUS*) decrease during the floral transition process in SF rose, whereas levels of *granule bound starch synthase 1* (*GBSS1*) and *starch synthase 2* (*SS2*) increase [8]. In apple, *TPS* displays a gradual increase during flower induction [48]. In addition, expression of sugar biosynthesis-related genes such as *SS2*, *GBSS*, and *SUS* increase during floral induction in *Litchi* and *Doritaenopsis* [49, 50]. Compared to ‘SX’, almost all DEGs related to starch and sucrose metabolism pathways were significantly altered during the T1 to T2 transition in ‘SJ’, whereas only 5 starch and sucrose-related DEGs changed significantly during the T2 to T3 transition. In contrast, almost all starch and sucrose-related DEGs in ‘SX’ were significantly altered during the T2 to T3 transition. This result suggests that starch and sucrose metabolism pathways might play vital roles in regulating the floral transition in these two longan genotypes, especially with regard to the emergence of floral primordia in PF longan cultivars. For example, starch synthase 2 and pgm, which are involved in starch metabolism, were only downregulated in the SJT1 and SJT2 comparisons. As a proxy for plant carbohydrate status, the signaling molecule trehalose-6-phosphate (T6P) plays a crucial role in the regulation of flowering [51]: TPS1 is necessary for normal vegetative growth and floral induction in *Arabidopsis*, and loss of TPS1 leads to extremely late flowering in *Arabidopsis*, even under other inducing environmental conditions [51]. However, in this study, *TPS1* was only significantly downregulated in ‘SJ’, indicating that *TPS1* may function as an inhibitor of PF traits in longan. Similarly, *GBSS1, SS2,* and *pgm* were also only downregulated in ‘SJ’. Conversely, the expression levels of several genes, such as *SUS* and *BAM4*, related to sugar biosynthesis were only significantly upregulated in ‘SJ’, indicating that these genes act as activators of PF traits in longan.

The flowering time of plants is determined by endogenous genetic components and various environmental factors, including temperature, stress, phytohormones, and day length [52]. Among these factors, hormone signaling plays a crucial role in the complex regulation of the floral transition [53]. A previous study has shown that changes in the contents of gibberellin (GA_3_), indole acetic acid (IAA) and zeatin ribosides (ZRs) differ during the natural floral differentiation process in ‘SJ’ and that ethephon and GA_3_ may promote flower formation and fruit setting in ‘SJ’ longan [54]. However, limited information is available on the effect of hormone signaling pathways on PF traits in plants. Auxin is an important hormone that participates in several aspects of plant growth and development, including floral induction [55]. In *Arabidopsis*, localized accumulation of auxin, which releases auxin response factor5/monopteros (ARF5/MP) from Aux/IAA repression, is the main reason for the emergence of flowers on the flanks of the shoot apical meristem (SAM) [56]. Similar to carbohydrates, the specific concentration of auxin is also a critical factor in floral induction because low auxin levels promote flowering and high concentrations delay flowering [52]. In addition, auxin biosynthesis and signaling pathway-related genes play an important role in flowering. For example, *Arabidopsis* mutants of ARF2, an auxin-mediated TF, display late flowering [57]. Levels of *GH3*, which maintains auxin homeostasis and promotes the conversion of auxin to amino acids, may increase during floral induction in SF rose and decrease in PF rose [8]. Similarly, in this study, many genes related to the auxin pathway, such as *GH3*, *LAX* (auxin influx carrier), *IAA* (auxin-responsive protein IAA), and *SAUR* family genes, were significantly differentially expressed during floral induction (T1 to T2 transition) in these two longan cultivars that exhibit differing flowering traits (Fig. 3 and Additional file 11). Therefore, these genes may participate in the promotion of PF traits through effects on the emergence of the floral meristem (FM).

The phytohormone cytokinin (CK) is a regulator of many processes in plants, including cell proliferation and differentiation, shoot and root growth, seed germination, and leaf senescence [58, 59]. Previous studies have reported that CK is involved in FM formation and that exogenously applied CK can promote flowering in *Arabidopsis* [60–63]. During the emergence of floral primordia, *Arabidopsis* histidine-containing phosphotransfer protein6 (*AHP6*), a negative regulator of CK signaling, is induced by the MP protein. AHP6 then may diffuse to adjacent sites, where it inhibits meristem initiation, thereby enhancing the auxin phyllotactic pattern [64, 65]. *Arabidopsis* histidine kinase2 (AHK2), AHK3, and AHK4/CRE1 are three membrane-located receptors that perceive the cytokinin signal [66–68], and lines carrying *ahk2* and *ahk3* variant alleles display an early flowering phenotype [58]. In the present study, most CK-related genes were significantly and differentially expressed during the floral induction process in both cultivars (Fig. 3 and Additional file 11). For example, 12 and 1 (Dlo_016152.1) *AHK* genes displayed significant up- or downregulation during the early floral induction stage in ‘SJ’, respectively, no significant changes were observed in ‘SX’, indicating these genes may participate in regulating floral induction in ‘SJ’.

GA is an important class of plant hormones involved in many aspects of development [69]. In woody species, a decline in GA is beneficial for floral induction [9, 70], and GA inhibits the floral transition in apple by repressing CK responses and signaling [71]. In this study, 7 gibberellin receptor *GID1* and 4 *DELLA* genes were upregulated in the early floral induction stage in ‘SJ’, and 3 *GID1* genes were downregulated. In contrast, 5 *GID1* and 2 *DELLA* genes were upregulated in the early floral induction stage in ‘SX’, and 2 *GID1* genes and one *DELLA* gene were downregulated. These results suggest that the GA signaling pathway may have different functions during floral induction in these two longan cultivars; however, further analyses are required.

Abscisic acid (ABA) also participates in many developmental processes such as seed development, stresses response, and floral transitions [72], and exogenous applications of ABA alters flowering time in several species [73]. In apple trees, ABA levels gradually increase in floral buds during the floral transition, indicating a positive role of ABA in flowering. Some ABA signaling-related genes such as *MYC2*, Pyrabactin resistance 1-like4 (*PYL4*), and *KIN10* (*SnRK2.6*) also gradually increase in floral buds during flower induction [71], and when exogenously applied to *Arabidopsis*, ABA functions as a inhibitor of flowering [74]. Similarly, the ABA level decreases during the floral transition in rose genotypes displaying SF or PF traits. Several ABA-related genes such as *PYL12* and *SnRK2/2.5* are downregulated, though *protein phosphatase 2C25* (*PP2C25*) is upregulated, during the floral transition [8]. In contrast, *PYL* genes were significantly upregulated but *PP2C* downregulated during the floral transition in ‘SJ’ and ‘SX’, indicating that the ABA signaling pathway may be involved in regulating different flowering traits in these two longan cultivars.

The steroid hormone brassinosteroid (BR), which is widely distributed in plants, plays a role in plant growth and development as well as responses to various stresses [75–77]. In addition, previous studies have reported that BR is involved in controlling the floral transition, and BR-deficient and -insensitive mutants are often described as late flowering [75, 78]. In *Arabidopsis thaliana*, *brassinosteroid-insensitive 1* (*bri1*) acts as an enhancer of the late flowering autonomous-pathway mutant *LUMINIDEPENDENS* (*LD*), and attenuation of BR signaling enhances *FLC* expression and delays flowering [79]. Similarly, *BSK* (Dlo_029469.1), *BZR1* (Dlo_010565.1), *CYCD3* (Dlo_000396.1 and Dlo_011212.2) and most *BRI1-like* genes were upregulated in the early floral induction stage in ‘SJ’ (Fig. 4 and Additional file 12), indicating that the BR signaling pathway may promote PF in longan. However, recent research indicates that the BR pathway TF BZR1 can upregulate FLC expression and consequent floral repression by recognizing and binding to a BRRE cis-element in the first intron of the gene [80]. Consistent with this result, levels of several *BRI1-like* genes decreased in the early floral induction stage in ‘SJ’.

Other hormone pathways such as the ethylene (ET), salicylic acid (SA), and methyl jasmonate (MeJA) signaling also affect floral induction [52, 81]. For instance, SA-deficient mutants exhibit late flowering under both long-day (LD) and short-day (SD) conditions by inducing expression of *FLC* [82]. MeJA appears to delay flowering in *Arabidopsis* and *Triticum aestivum*, and mutation of *bHLH TF* genes represses JA signaling, resulting in late blooms; the JA receptor mutant *coi1* also shows early flowering [83, 84]. Mutants of Ser/Thr kinases *CTR1* (*Atctr1*), which act as inhibitors of ethylene signaling, exhibit late flowering, whereas mutations in *OsERS2*, *OsETR2*, and *OsETR3* cause enhanced ethylene sensitivity and early flowering, indicating that ethylene inhibits flowering in *Arabidopsis* and rice [85, 86]. In the present study, *CTR1*, jasmonic acid-amido synthetase *JAR1*, *MYC2*, and *bHLH TF* genes were upregulated during early floral induction in ‘SJ’, and *ERF-1* (ethylene response factor) and *TGA1 TF* genes were downregulated during early floral induction in both ‘SJ’ and ‘SX’. In summary, hormone biosynthesis and signaling pathways may affect PF trait formation in longan. However, further research is needed on the specific functions of hormone signaling in regulating PF trait formation in longan because of the complex networks of crosstalk between different hormones and reported contradictions between various species.

Flowering is a complex process of morphogenesis that is controlled by a complex network involving vernalization, autonomous, photoperiod, GA-dependent, and aging pathways. These pathways, which are both independent and cross-linked, generate positive and negative feedback and combine flowering signals into several key floral integrators (e.g., FT, TSF and SOC1), activate floral meristem identify genes (e.g., *LFY* and *AP1*), and promote flowering [52, 87, 88]. The genetic control of PF has been studied in several model plants such as *Arabidopsis*, strawberry and rose [16–20], though the complex regulatory mechanisms involved in PF trait formation in fruit trees are still unknown. Although most flower-related genes are conserved among species [89, 90], the function of many of these genes differ between model plants and woody plants [15]. For instance, *TFL1* and *FLC*, which control PF in *Arabidopsis* and strawberry showed no differential expression during the floral induction process in our study, possibly indicating a different mechanism of PF trait formation between longan and model plants. Thus, it is necessary to investigate the regulatory mechanisms involved in PF trait formation in fruit trees, which is directly linked to production potential. In this study, 39 flower-related genes differentially expressed during floral induction between ‘SJ’ and ‘SX’ were identified (Fig. 5 and Additional file 13). Most are associated with the photoperiod and circadian clock pathways, such as the *COL* gene, *APRRs*, *FKF1*, phytochrome, and TF TCPs, indicating that photoperiod and circadian clock-mediated floral induction are essential for determination of the floral fate in ‘SJ’ longan. Surprisingly, no clear differential expression of genes associated with vernalization and autonomous pathways, which might be important for fruit trees, was found during floral induction between the two longan cultivars. *CO* acts as a network hub, integrating various external and internal signals into the photoperiod and circadian clock pathways [91]. As a key gene in the photoperiod pathway, *CO* controls the floral transition by directly inducing expression of the *FT* gene [92]. The transcriptional level of *CO* is affected by *GIGANTEA* (*GI*), which participates in the circadian clock pathway [93, 94]. The GI-CO-FT module is the main photoperiod and circadian clock pathways in *Arabidopsis* and also exists in other plants such as in *Populus deltoides* and apple [71, 95]. In this study, three *CO-like* genes (*COL2*, Dlo_003578.1; *COL7*, Dlo_005461.1; *COL10*, Dlo_013961.1) were upregulated during the floral induction process in ‘SJ’, whereas *GI* (Dlo_024864.1) displayed an opposite expression pattern, suggesting that these *COL* and *GI* genes may function as activators or suppressors of floral induction in ‘SJ’. Similar to *COL* genes, two teosinte branched 1/cycloidea/proliferating cell nuclear antigen factor (*TCP*) genes (*TCP4-like*, Dlo_026743.1; *TCP5-like*, Dlo_025323.1), which act as CO activators [96], and the *GAI* gene were upregulated during floral induction in ‘SJ’. Pseudo-response regulator (PRR) proteins have the ability to increase CO binding to the FT promoter, leading to FT transcriptional enhancement and early flowering [97]. In the present study, 5 *PRR* genes showed changes similar to those of *GI*, indicating potential functions in PF trait formation in longan. Early flowering (EFL) proteins are a type of circadian clock component with an important role in flowering. For instance, EFL3 inhibits flowering under noninducing photoperiods by blocking the production of GA and expression of *FT1* [98]. Jia et al. (2014) found that the expression level of *ELF4* (Unigene4309) increased in ‘SJ’ compared with ‘Lidongben’, indicating that *ELF4* may be involved in PF traits and that *ELF4* may be a key gene. Similarly, the *EFL4-like* gene (Dlo_027544.1) functions as a suppressor of floral induction in ‘SJ’. APETALA2 (AP2), a target of miR172 that has been implicated in floral stem cell control [99], was downregulated during floral induction in ‘SX’, as was *COL14*, indicating a promoting role in this process. Guo et al. [8] reported similar results. *LFY*, a plant-specific TF, is the central floral meristem identity gene [100], and the expression level of *LFY* is an important determinant of flower initiation [101]. *LFY* acts as a key regulator in the integration of flowering signaling pathways, controls the transition from the inflorescence meristem to the floral meristem, and regulates flowering time [62]. During the emergence of floral primordia, *LFY* is directly induced by the MP protein, and it then activates expression of the downstream regulator axillary meristems1 (*RAX1*) and simultaneously inhibits expression of *Arabidopsis* response regulator7 (*ARR7*), thereby activating CK signaling and eventually promoting floral meristem formation [102–104]. In the young floral meristem, the MADS-box genes cauliflower (*CAL*), short vegetative phase (*SVP*), agamous-like 24 (*AGL24*), and apetala1 (*AP1*) are induced by *LFY* to specify floral identify. Indeed, a feed-forward regulatory loop between *AP1* and *LFY* stabilizes floral identity and inhibits *TFL1* to promote the transition from the inflorescence meristem to the floral meristem [62]. However, a recent study showed that in the absence of AP1/CAL activity, *TFL1* expression is suppressed by *AP1* and directly promoted by *LFY*, indicating that *LFY* has an inhibitory effect on flower formation [105]. Consistent with this study, *LFY* (Dlo_005438.1) was significantly upregulated during T1 to T2 in ‘SX’, though it showed no apparent changes during flower induction in ‘SJ’, suggesting a potential inhibition role in longan PF trait formation.

In addition to the above genes involved in different pathways, several TFs such as GRAS, MADs, NAC and MYB also play a vital role in regulating downstream floral transition genes [36–39]. Previous research showed that some GRAS family genes may be involved in regulating the floral transition in species such as grape and apple [39, 106]. In our study, 52 GRAS genes were found in the longan genome, and 19 GRAS TFs displayed differential expression during flower induction in ‘SJ’ and ‘SX’ (Fig. 6 and Additional file 14). Interestingly, among these 19 GRAS TFs, two RGL1-like genes (Dlo_024980.1 and Dlo_008157.1) and one GAI (Dlo_019465.1) gene are involved in the GA-mediated signaling pathway. Moreover, DELLA proteins may repress the transcriptional activity of the CO TF by direct interaction, and RGL1 is likely involved in sugar metabolism in SF rose [69]. We also identified 109 MADS-box family genes in the longan genome. MADS-box family genes are also involved in plant floral induction; for example, the *AGL6* gene (*OsMADS6*) determines floral meristem and floral organ identity in rice [36]. Among them, 15 of these genes displayed differential expression during flower induction in ‘SJ’ and ‘SX’ (Fig. 6 and Additional file 14). NAC participates in floral induction in several species [37, 107], and in longan, 31 of 107 NAC-like family members were differentially regulated during flower induction in ‘SJ’ and ‘SX’ (Fig. 6 and Additional file 14). Seven genes (Dlo_028054.1, Dlo_012365.1, Dlo_022129.1, Dlo_012309.3, Dlo_028436.1, Dlo_020074.1 and Dlo_005893.1) were common, and expression of the remaining genes was significantly altered during flower induction in ‘SJ’ and ‘SX’. MYB TFs are also essential for floral organ development and play key roles in pollen development in various species such as cotton and *Arabidopsis* [38, 108]. MYB levels decrease in PF rose during floral transition [8]. Consistent with these results, 19 and eight genes showed significant up- and downregulation, respectively, during T1 to T2 in ‘SJ’, and 17 genes displayed differential expression during flower induction in ‘SX’ (Fig. 6 and Additional file 14). Hence, these TFs may participate in the formation of longan PF traits.

## Conclusion

Research to date on the regulatory mechanisms involved in PF has mainly focused on model plants. As an important trait that is directly linked to production potential, it is necessary to elucidate the regulatory mechanisms involved in PF trait formation in fruit trees. In this study, comparative transcriptome analysis was performed using two longan cultivars that exhibit differing flower phenotypes during the floral induction process. According to our study, the transcriptional differences in ‘SJ’ are mainly concentrated at the early floral induction stage, which is consistent with a previous study showing that red buds (T2 in our study) are the signal for floral induction. Comparing these two longan genotypes, the transcriptional landscape of the T1 to T2 transition differed greatly with regard to key hormones, circadian rhythm, and sugar pathways, as well as TFs. Almost all of the flowering-related DEGs identified are involved in the photoperiod and circadian clock pathways. Unexpectedly, *TFL1* and *FLC*, which control the PF trait in *Arabidopsis* and strawberry, showed no significant differential expression during floral induction in our study, possibly indicating a novel mechanism of PF trait formation in ‘SJ’ longan. In addition, the *LFY* gene may inhibit PF in ‘SJ’ longan, though further analyses are required. This study provides a platform for understanding the molecular mechanisms underlying changes between PF and SF longan genotypes and will aid future studies on PF trait mechanisms of evergreen fruit trees.

## Additional files

Additional file 1: List of primers used in this study. (XLSX 12 kb)

Additional file 2: The sequencing and mapped results obtained from each sample. (XLS 22 kb)

Additional file 3: Identification of known genes and new genes in the transcriptome data for eighteen samples. (XLSX 11 kb)

Additional file 4: DEGs between ‘SJ’ and ‘SX’ during floral induction. (XLS 11846 kb)

Additional file 5: Significantly enriched GO term analysis of DEGs between ‘SJ’ and ‘SX’ during floral induction. (XLSX 22 kb)

Additional file 6: Significantly enriched KEGG pathway analysis of DEGs between ‘SJ’ and ‘SX’ during floral induction. (XLSX 15 kb)

Additional file 7: DEGs in ‘SJ’ during floral induction. (XLS 7620 kb)

Additional file 8: DEGs in ‘SX’ during floral induction. (XLS 7771 kb)

Additional file 9: GO terms significantly overrepresented in the eight enriched profiles of gene expression versus the reference set in ‘SJ’ and ‘SX’. (XLSX 71 kb)

Additional file 10: KEGG pathways significantly overrepresented in the eight enriched profiles of gene expression versus the reference set in ‘SJ’ and ‘SX’. (XLSX 20 kb)

Additional file 11: Sugar-related DEGs in two longan cultivars during the floral induction process. Red indicates the genes that showed upregulated expression; blue indicates the genes that showed downregulated expression. (XLSX 48 kb)

Additional file 12: Hormone-related DEGs in two longan cultivars during the floral induction process. Red indicates genes that showed upregulated expression; blue indicates genes that showed downregulated expression. (XLSX 107 kb)

Additional file 13: Flower-related DEGs in two longan cultivars during the floral induction process. Red indicates genes that showed upregulated expression; blue indicates genes that showed downregulated expression. (XLSX 26 kb)

Additional file 14: GARS, NAC, MADs, MYB TFs in two longan cultivars during the floral induction process. Red indicates genes that showed upregulated expression; blue indicates genes that showed downregulated expression. (XLSX 125 kb)

Additional file 15: Linear relationships between qRT-PCR data and RNA-Seq data of related genes. The x-axis indicates the qRT-PCR log_2_ expression ratios; the y-axis indicates the RNA-Seq data ratios. (TIF 262 kb)

## Abbreviations

DEG: Differentially expressed gene
FDR: False discovery rate
GO: Gene Ontology
KEGG: Kyoto Encyclopedia of Genes and Genomes
NCBI: National Center of Biotechnology Information
qRT-PCR: quantitative real-time reverse transcription polymerase chain reaction
RNA-Seq: RNA sequencing
RPKM: Reads per kilobase of exon model per million mapped reads
