# Supplementary figures and images for: Comprehensive analysis of the longan transcriptome reveals distinct regulatory programs during the floral transition
Source: BMC Genomics. 2019 Feb 11;20:126. doi: 10.1186/s12864-019-5461-3 (PMC6371577; doi:10.1186/s12864-019-5461-3)

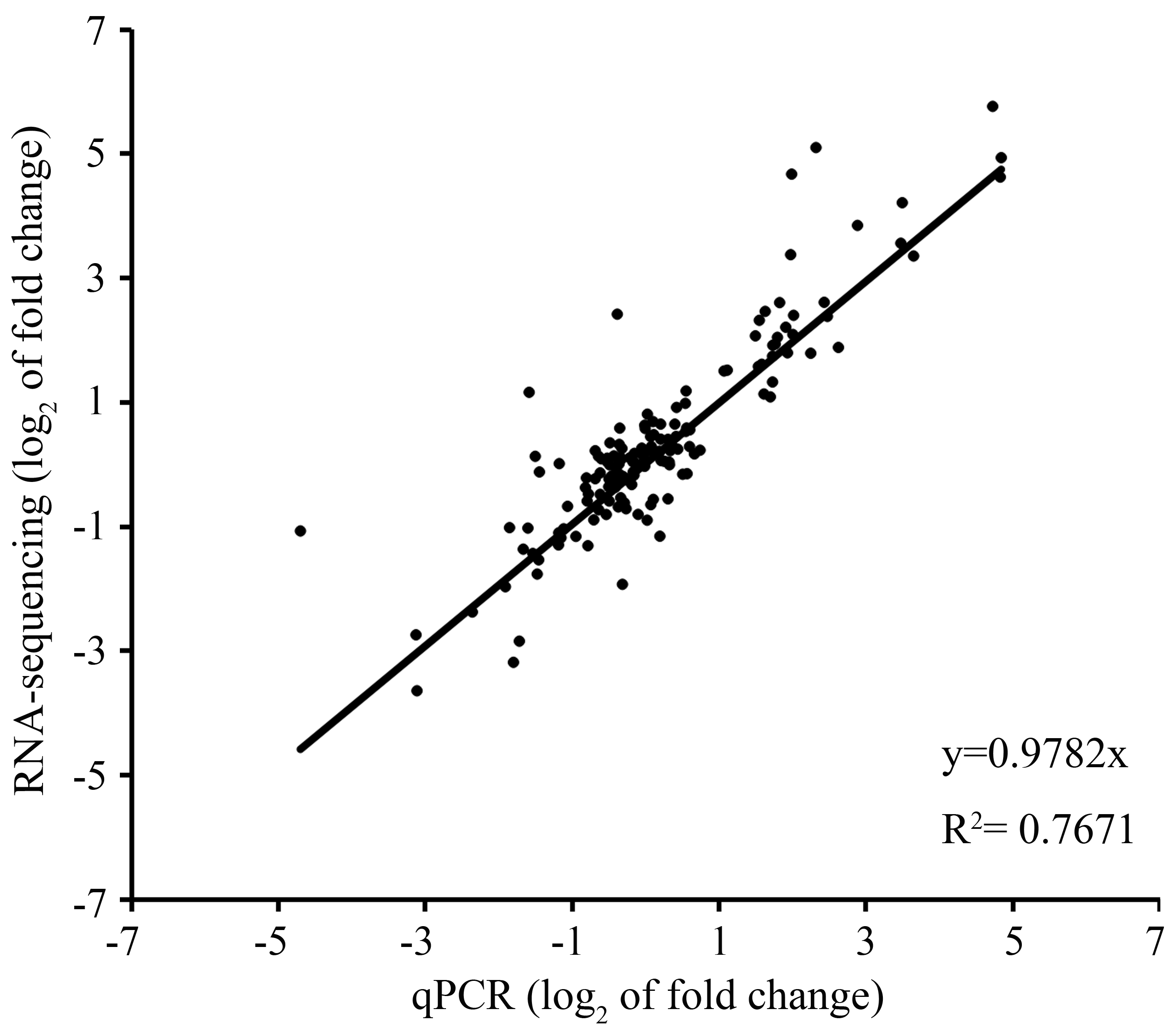

Supplement: Supplementary file 15 — Linear relationships between qRT-PCR data and RNA-Seq data of related genes. The x-axis indicates the qRT-PCR log2 expression ratios; the y-axis indicates the RNA-Seq data ratios. (TIF 262 kb) [file 12864_2019_5461_MOESM15_ESM.tif]
